# Molecular Techniques as Alternatives of Diagnostic Tools in China as Schistosomiasis Moving towards Elimination

**DOI:** 10.3390/pathogens11030287

**Published:** 2022-02-24

**Authors:** Chao Lv, Wangping Deng, Liping Wang, Zhiqiang Qin, Xiaonong Zhou, Jing Xu

**Affiliations:** 1 National Institute of Parasitic Diseases, Chinese Center for Disease Control and Prevention (Chinese Center for Tropical Diseases Research), NHC Key Laboratory of Parasite and Vector Biology, WHO Collaborating Centre for Tropical Diseases, National Center for International Research on Tropical Diseases, Shanghai 200025, China; lvchao@nipd.chinacdc.cn (C.L.); dengwp@nipd.chinacdc.cn (W.D.); qinzq@nipd.chinacdc.cn (Z.Q.); 2 School of Global Health, Chinese Center for Tropical Diseases Research, Shanghai Jiao Tong University School of Medicine, Shanghai 200025, China; 3 Jiading District Center for Disease Control and Prevention, Shanghai 200025, China; wlpnlnl@163.com

**Keywords:** schistosomiasis japonica, elimination, diagnostic tools, molecular techniques

## Abstract

Schistosomiasis japonica caused by the trematode flukes of *Schistosoma japonicum* was one of the most grievous infectious diseases in China in the mid-20th century, while its elimination has been placed on the agenda of the national strategic plan of healthy China 2030 after 70 years of continuous control campaigns. Diagnostic tools play a pivotal role in warfare against schistosomiasis but must adapt to the endemic status and objectives of activities. With the decrease of prevalence and infection intensity of schistosomiasis in human beings and livestock, optimal methodologies with high sensitivity and absolute specificity are needed for the detection of asymptomatic cases or light infections, as well as disease surveillance to verify elimination. In comparison with the parasitological methods with relatively low sensitivity and serological techniques lacking specificity, which both had been widely used in previous control stages, the molecular detection methods based on the amplification of promising genes of the schistosome genome may pick up the baton to assist the eventual aim of elimination. In this article, we reviewed the developed molecular methods for detecting *S. japonicum* infection and their application in schistosomiasis japonica diagnosis. Concurrently, we also analyzed the chances and challenges of molecular tools to the field application process in China.

## 1. Introduction 

The worldwide pandemic of COVID-19 made administrators of government and all residents aware of the enormous threat from infectious disease to public health safety and social–economic growth [1,2]. Being a widespread tropical disease, schistosomiasis is endemic in 78 countries, with at least 236.6 million people required preventive treatment in 2019 according to the data from WHO. There are three major types of schistosomiasis affecting human beings: schistosomiasis japonica, schistosomiasis mansoni, and schistsomiasis hamatobium. Schistosomiasis japonica caused by *Schistosoma japonicum* (*S. japonicum*) is distributed in China, the Philippines, and small pockets of Indonesia in Asia, whereas the other two kinds of schistosomiasis, caused by *S. haematobium* and *S. mansoni*, are mainly distributed in countries belonging to Africa and South America [3,4]. Schistosomes are remarkable parasites for being exquisitely adapted to their two-host life cycle, which involves a short period of substantive population booms in the molluscan intermediate host and a long sheltered life, including prodigious egg laying in definitive mammalian hosts [5]. Based on the insight and experience that blocking any stage of the life cycle of *S. japonicum* would interrupt the transmission, the detection of individual or worm carriers and infected snails with the intention of identifying sources of infection and risk environments is extremely vital in control activities [6,7].

Applying optimal methodologies for diagnosis adapted to the changing control situation is crucial to every aspect of schistosomiasis control. Parasitological tests, such as the miracidia hatching technique (MHT) and Kato–Katz method (KK), are recommended as the “gold” standard for schistosomiasis diagnosis, especially in situations with high prevalence, but they are highly likely to miss infection with light intensity from low prevalence areas [8,9]. That is to say, the insensitivity of etiological detection will lead to an underestimation of disease burden, even threatening the success of the national program and the ultimate goal of elimination [10]. Serological tests based on the immunological response of antigens and antibodies have been widely applied in preliminary screening of patients and disease surveillance in many endemic countries with schistosomiasis, owing to advantages such as low cost, ease of operation, independence of complicated and expensive equipment, etc. [11,12]. However, serological methods have often been criticized for being relatively non-specific, prone to cross-reaction with other parasitic diseases, and unable to distinguish past from current infections [13,14]. With the flourishing of gene and genome research, nucleic acid detection provides a new idea for the diagnosis of schistosomiasis, presenting advantages of higher sensitivity and overwhelming specificity over immunological tests [13,15]. They are also superior in identifying infection by different species of *Schistosoma* in cases of low-grade infection [16,17]. Better yet, nucleic acid detection-based methods could complete detection based on various samples, including snail tissue [18,19], environmental samples [20], sera, and stool [21,22]. Although most molecular techniques still require expensive infrastructure and highly accurate pretreatment of samples, such as DNA extraction, they are the most potential and valuable methods used for disease surveillance and verifying elimination in many countries with very low endemicity of schistosomiasis, especially with the development of isothermal amplification techniques [23,24]. 

In this paper, we reviewed the existing diagnostic methods specially focused on schistosomiasis japonica, emphatically introducing the progress of nucleic acid detection-based methods. We also analyzed the potential of the molecular techniques applied in the national control program and the challenges that exist to provide reference for related experts of diagnosis and policymakers. 

## 2. Schistosomiasis Japonica and the Agenda of China

Schistosomiasis japonica is a zoonotic disease, with human beings and more than 40 mammalian definitive hosts identified thus far [3,4]. Although it is endemic in China, the Philippines and Indonesia, the strain of *S. japonicum* in China is more virulent, with a wide range of epidemic areas and a heavier disease burden than the parasite in the other two countries [25]. Schistosomiasis japonica was a great public health threat in the 1950s with around 12 million infected people mainly distributed in 12 provinces of China along the Yangtze River [26,27,28], where the climate and environment are highly suitable for the propagation of oncomelanade snails. Slightly different from the WHO NTD roadmap’s three time-bound goals for the control or elimination of schistosomiasis, the control process was divided into four stages or three criteria, in order: infection control (prevalence < 5% in humans and animals), transmission control (prevalence < 1% in humans and animals), transmission interruption (zero infection in local residents, domestic animals and snails in five consecutive years), and elimination (zero infection in local residents, domestic animals and snails in another five consecutive years after transmission interruption) [29,30,31].

Nowadays, schistosomiasis japonica has been eliminated in many previous endemic regions due to the high priority over schistosomiasis control at the political level, nearly 70 years of continuous national control program and the effort of multi-sectoral experts and local residents, as well as the flourishing development of economy and technology in China [10,32,33,34]. Five provinces, Shanghai, Guangdong, Guangxi, Fujian, and Zhejiang, have been pioneers in schistosomiasis elimination [35,36]. In 2019, only 5 cases (5 out of 327,475) and 7 domestic animals (7 out of 134,978) were found to be etiologically positive nationwide [37]. By 2020, only 15 of 450 previous endemic counties were maintained in the stage of transmission control due to their complicated environment and relatively retarded economy. However, interrupting the transmission and eventually eliminating schistosomiasis completely nationwide, which set the national strategic plan of Healthy 2030, has turned out to be difficult [33,38]. To accelerate the process of schistosomiasis elimination, optimal methodologies with high sensitivity and absolute specificity are needed for the detection of asymptomatic cases or light infections, as well as disease surveillance to verify elimination.

## 3. Traditional Diagnostic Tools Applied in Schistosomiasis Control in China

It cannot be exaggerated that diagnosis is the essential basis of schistosomiasis control for case identification and treatment, assessment of morbidity, and evaluation of control strategies, which are all dependent on the performance of diagnostic tests [27,39]. Two kinds of diagnostic methodologies, namely parasitological techniques mainly including KK and MHT [40,41], and immunologic approaches based on detection of specific antibodies, were widely used in the national control program in China, accelerating the process of schistosomiasis control significantly [11,42]. 

### 3.1. Parasitological Methods

The KK method, which was originally developed in the mid-1950s, and MHT, based on the positive phototactic behavior of miracidia, are the most broadly used techniques in epidemiological surveys pertaining to schistosomiasis in China [43]. At the stage of morbidity control, especially in the 1950–1980 period, the parasitological methods were the most applicable to the field, featured by high prevalence and high infection intensity. In 1989, with the first national survey of schistosomiasis japonica, direct stool examination of the KK method or MHT was still the recommended method to evaluate the prevalence of schistosomiasis [6,43]. Until now, the KK method and MHT have been the most accepted “gold” standard methods for identifying whether people or animals are infected [6,44]. Additionally, some studies indicated that multiple KK tests per sample, or increasing the collection frequency of stool samples, would increase the diagnostic sensitivity [45,46,47], and the MHT technique possessed higher sensitivity than the KK method due to the larger volume of stool tests received. Additionally, the combination of MHT and microscopic examination of filtered stool sediment would increase the detection rate [48]. 

### 3.2. Immunologic Tests

The immunological tests for schistosomiasis diagnosis also have a long history in China. The earliest immunologic test in China was the intradermal text (ID) recorded in 1936, and it was adapted for screening prior to further parasitological examination in the national general survey in the 1950s. Subsequently, a variety of immunologic techniques were applied to the national schistosomiasis control program, such as the circumoval precipitin test (COPT), indirect hemagglutination assay (IHA), the enzyme-linked immunosorbent assay (ELISA), and some rapid diagnostic tests (RDTs). Nowadays, ID and COPT have been out of use due to their low specificity, while IHA is still widely used in most endemic areas of China. Currently, there are two IHA kits with high quality control accredited by the China Food and Drug Administration. In the 10-year World Bank Loan Project (WBLP), aiming to control the morbidity of schistosomiasis, immunological tests were used directly to determine the target of chemotherapy in areas of medium endemicity (15% > prevalence > 3%) and low endemicity (prevalence < 3%) [43]. In the second national survey of schistosomiasis in 2004, in China, the ELISA method was adopted as a screening tool followed by stool examination to understand the real infection status of schistosomiasis in human beings. With the achievement of infection control reached in 2008 and transmission control reached in 2015, a diagnostic strategy with primary immunodiagnostic screening followed by KK or MHT for antibody-positive individuals was widely used in the Chinese national control program and routine surveillance activities in sentinel sites [27]. 

## 4. Molecular Methods Developed to Detect the Pathogen of Schistosomiasis 

**Figure 1:**
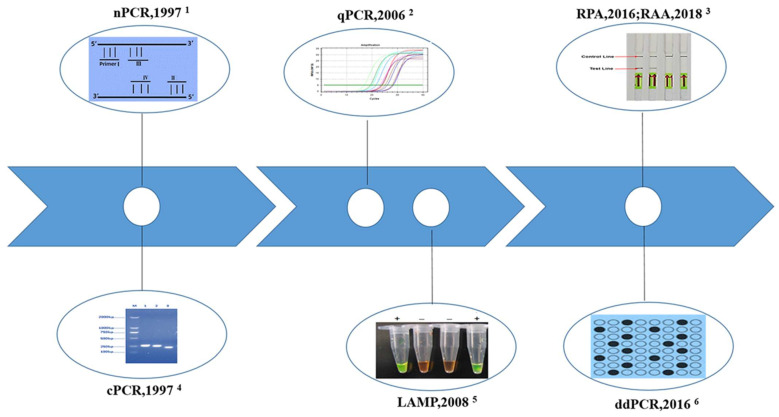
The molecular diagnostic techniques of schistosomiasis japonica and the year of first report in China. ^1^ nPCR is characterized by two pairs of primers, inner primer and outer primer; ^2^ qPCR can quantitatively and qualitatively analyze the initial template of samples by detecting the fluorescence signal corresponding to each cyclic amplification product in real time; ^3^ The combination of RPA and lateral flow dipstick (LFD) for visual detection. Generally, visualization of control line and the test line is positive, and only the control line is negative; ^4^ cPCR determined positive or negative results by the size of the gel electrophoresis band; ^5^ The combination of LAMP reaction with chemical dyes for visual detection. + positive reaction, − negative reaction; ^6^ Dilute sample or samples DNA to the single molecule level and collect the fluorescence signal of a single reaction unit to achieve the absolute quantitative detection. Black spots: positive reaction units, blank spots: negative reaction units.

With the goal of the national program shifting from the control of schistosomiasis to elimination, extremely sensitive and specific diagnostic tools are needed emergently to explore asymptomatic cases with light infection and verify transmission interruption or elimination [34,49]. With the development of genomics and genome data for parasites and the urgent demands, molecular diagnostic techniques based on nucleic acid detection have emerged as new hot spots [50]. Various polymerase chain reaction (PCR)-based parasite DNA detection assays, including conventional polymerase chain reaction (cPCR), nested PCR (nPCR), real-time quantitative PCR (qPCR), and droplet digital PCR (ddPCR), have stimulated much interest as alternative options due to their proven diagnostic accuracy and the ability to detect early pre-patient infections [51,52]. The emergence of isothermal amplification methods, such as loop-mediated isothermal amplification (LAMP) and recombinase polymerase amplification (RPA), solves the dilemma of costly instrument dependence on PCR-based methods. The molecular diagnostic techniques of schistosomiasis japonica and the year first reported in China are shown in Figure 1. Regrettably, we have not seen any product based on molecular techniques applied in the field on a large scale.

### 4.1. Conventional Polymerase Chain Reaction (cPCR)

cPCR emerged in the 1980s with a specific ability to amplify a small amount of target DNA and was the first nucleic acid amplification test used in schistosomiasis japonica diagnosis [53]. The character of amplification of microscale DNA of different samples greatly improves the analytical ability, simplifies the diagnostic process, and increases the sensitivity [14,16,51]. Another obvious advantage of this technology is that the amplified products can be visualized by gel electrophoresis and verified by sequencing. So far, many cPCR methods for the diagnosis of schistosomiasis japonica targeting various genes from chromosomes and mitochondria extracted from different samples have been established (Table 1). One of the most important factors impacting the sensitivity of cPCR or other types of molecular detection assays is the abundance of the target sequences or biomarkers in the chromosome or mitochondrial genome [16,51]. The earliest reported PCR test for schistosomiasis diagnosis in China was based on the gene coding miracidium antigen named Sj5D [54,55,56]. Nowadays, the highly repetitive and conserved subunits of 18S rRNA [57,58,59,60], 28S rRNA [18,61,62,63], cytochrome c oxidase subunit 1 (COX I) [64,65], and the repetitive sequences SjR2 (an RTE-like, non-long terminal repeat retrotransposon from *S. japonicum*) [18,21,66,67,68,69] were the most common detection biomarkers. To evaluate the efficiency of established cPCR methods, several kinds of specimens, including genomic DNA (gDNA), pooled samples of snail and cercaria, and a mixture of eggs and feces, were employed. The lowest detection limit of cPCR established targeting a 607 base pair (bp) region of COX I can reach 10 fg of gDNA, which is less than that of 1 egg or 1 cercariae [64].

cPCR also provided a potential tool for the early detection and therapy evaluation of *S. japonicum* infection. The cPCR assay using a 230-bp sequence of SjR2 established by Xia could detect *S. japonicum* DNA in sera at the first week post-infection, and it became negative at 10 weeks post-treatment in a rabbit model infected by *S. japonicum* [21]. Similarly, schistosome DNA can be detected from one day post infection using pooled urine samples of mice by COX I-cPCR [65]. Moreover, the cPCR assay was always used as a reference method to assess the efficacy of other established methods, not only molecular methods but also serological methods [60,70,71]. The cPCR assay targeting 254-bp size of COX I gene was used in the detection of human samples in highly endemic areas of the Philippines, and the results showed that schistosome DNA in the serum and urine of KK-positive subjects could be detected by COX I -cPCR with 100% sensitivity [65]. However, few studies have reported on detecting field samples by the cPCR method, which may largely be due to its dependence on specific equipment and relatively complicated producers.

**Table 1:** The target genes and sample types of established cPCR methods for schistosomiasis japonica.

	Target Sequence	No. of GenBank Accession	Fragment Size (bp)	Detection Limit	Specimen	Year of Publication
1	Sj5D	N/D ^1^	262	1 cercarial; 1 egg	Animal tissue and blood	1997,1998 [54,55]
2	Sj5D	N/D	262	10 fold diluted single cercarial DNA	Cercarial DNA	2004 [56]
3	18S rRNA	DQ442999	469	40 pg	gDNA	2006 [57]
4	18S rRNA	DQ442999	469	1 cercaria	Cercaria	2008 [58]
5	18S rRNA	DQ442999	463	62.5 pg	gDNA	2010 [59]
6	18S rRNA	FJ176682	157	1 cercaria in pooled 10 non-infected snails; 2 eggs in 100 mg of non-infected fecal sample	Snail; Fecal of mice	2013 [60]
7	28S rRNA	Z46504	405	100 fg	gDNA	2010 [18]
8	28S rRNA	Z46504	607	15 pg	gDNA	2006 [61]
9	28S rRNA	Z46504	200	N/D	Cattle fecal	2017 [62]
10	28S rRNA	EU835689.1	330	N/D	Snail	2018 [63]
11	COX1	AF215860	614	10fg	gDNA	2010 [64]
12	COX1	AF215860	254	N/D	Serum and urine	2015 [65]
13	SjR2	AF412221	230	1 pg	gDNA	2010 [18]
14	SjR2	AF412221	230	0.8 pg	gDNA	2009 [21]
15	SjR2	N/D	176	1 cercariae	Cercariae	2005 [66]
16	SjR2	AF412221	230	0.021 eggs	Egg DNA	2007 [67]
17	SjR2	AF412221	230	0.5 eggs/g of feces	Human feces	2012 [68]
18	SjR2	N/D	408	1 egg	Colon tissue	2019 [69]
19	Mitochondrial DNA gene	N/A ^2^	668/242	0.3 eggs	Fecal of mice	2005 [72]

^1^ N/D: Non-disclosed; ^2^ N/A: Non-applicable.

### 4.2. Nested PCR (nPCR)

The assay of nPCR, which can be considered a variant of cPCR, requires two rounds of PCR amplification using two sets of primers, commonly called the outer primers and the inner primers [17]. nPCR is more sensitive and specific than cPCR because the probability is extremely low if the first round amplification produces an erroneous fragment; primer pairing and amplification will occur in the second round amplification using the wrong fragment [51]. The repetitive sequences of SjR2 and SjCHGCS19 (a new 303-bp sequence from non-long terminal repeat (LTR) retrotransposon) are the common biomarkers in nPCR assays [73,74]. The published literature showed that the detection limit of SjR2-nPCR stabilized at fg level with 10fg of minimum limit [75]. Validated by the schistosome-infected mice model [76], rabbit model [74,77], and domestic animals (goat and buffalo) [78], the SjR2-nPCR or SjCHGCS19-nPCR could all be used for early diagnosis of schistosomiasis, even light infection, showing positive results at 3 days post-infection through testing sera samples. For samples from humans with chronic schistosomiasis, the detection rate of 230-bp SjR2-nPCR assay was 88.79% (95/107), significantly higher than that of the KK method (69.16%, 74/107) [79]. The sensitivity of the SjR2-nPCR method established by Zhang et al. using 14 and 28 days post-infection buffalo samples was 92.30% (36/39) and 100% (39/39), while the specificity was 97.60% (41/42) [78]. Moreover, the SjCHGCS19-nPCR assay demonstrated 97.67% sensitivity for 43 patient serum samples and 96.07% specificity for 51 serum samples from healthy individuals [74]. In addition, an nPCR assay targeting the 420-bp fragment of the Sjα1 gene, which is a short dispersal element retrotransposon gene with high copy and expression throughout the life cycle of schistosomes, could detect 0.1 fg of gDNA and distinguish the infection status of snail 4h post-infection [80]. However, the impressive performance of the detection efficacy of nPCR needed more verification.

### 4.3. Real-Time Quantitative PCR (qPCR)

The technique of the qPCR assay uses fluorescent labeled probes or double-stranded DNA-specific fluorescence dye to enable the continuous monitoring of amplicon (PCR product) formation throughout the reaction, thus allowing the quantification of PCR products by measuring fluorescence [81,82]. Compared with cPCR, qPCR has the following advantages: the amount of DNA in a sample can be measured using a standard curve, which can be determined by either spiking samples with a known amount of template or serial DNA dilutions; the qPCR procedures are streamlined with no need for an additional electrophoresis step to detect end-products of PCR, and the results can be preserved for a long time; and qPCR can utilize multiplex assays to detect multiple infections within a single clinical sample using specific probes and is preferred over cPCR in multiplex assays for having improved specificity by the use of probes [51,83].

The first reported qPCR assay for the detection of *S. japonicum* appeared in 2006, with mitochondrial NADH I as the target gene, and its detection limit could reach 1 egg per gram (EPG) fecal [84]. In the detection of 1,727 persons in field settings of Anhui Province, China, the prevalence (no. positive/no. examined) determined by NADH I-qPCR was 5.3%, significantly higher than those of the hatching test (3.2%) and Kato-Katz thick smear (3.0%) [85]. In a field evaluation of qPCR assay conducted in Hunan, Anhui, Hubei, and Jiangxi provinces of China, the qPCR assay exhibited a high level of sensitivity (100% for humans, 96.83% for bovines) and specificity (100%), and obtained a significantly higher prevalence in both the human (11.06% for qPCR, 0.93% for MHT) and bovine samples (24.73% for qPCR, 7.69% for MHT) [86]. The research conducted in the Philippines also showed similar trends, demonstrating that traditional copro-parasitological techniques underestimate the infection rate, signifying the advantages of qPCR for case finding and disease surveillance and monitoring [87,88,89,90,91]. Besides the gene of NADH I [85,86,87,88,89,90,91,92,93,94,95], several qPCR methods have been established targeting other genes, including COX I [96], 18S rRNA [97,98,99,100,101], ITS 2 [102], SjR2 [99,103,104], SjCHGCS20 [22], SjCHGC08270 [20,105], and Sjrh1.0 [99,106] (Table 2). Those established qPCR methods have mostly completed the laboratory evaluation, but further validation should be conducted in field settings. Notably, the TaqMan qPCR assay targeting the COX I gene was developed to detect the environmental DNA (water samples) of *S. japonicum*, and its potential utility to schistosomiasis japonica surveillance in the Philippines was assessed. The results showed that the qPCR method could complement malacological surveys for monitoring schistosomes in endemic areas, especially those with a high risk of human infection [96].

### 4.4. Droplet Digital PCR (ddPCR)

The technique of ddPCR can still be considered a ‘new’ technology in parasitology, including schistosomiasis. Owing to its sensitivity and absolute quantitative characteristics, ddPCR is a potential candidate to become an appealing new method for parasite detection and quantitative analysis in the future [107]. Weerakoon et al. developed a ddPCR duplex assay targeting SjR2 and NADH I for the detection of *S. japonicum*, which provides improved detection sensitivity and specificity. The assay was able to detect as little as 0.05 fg of template DNA, and exhibited a high sensitivity for the detection of low levels of parasite DNA in stool, serum, urine, and saliva of mice model [108,109]. The ddPCR assay was also validated using clinical samples collected from 412 residents in a moderate-endemic area of schistosomiasis in the Philippines, proving its higher level of sensitivity obtained for human stool, serum, urine and saliva samples compared with the microscopy-based KK test [110,111]. Moreover, the capacity of ddPCR to quantify infection intensity has important public health implications for schistosomiasis control. Van Dorssen et al. determined the infection prevalence of *S. japonicum* in fecal samples of goats using the gene NADH I (46.4% ddPCR vs. 6.9% qPCR), showing that ddPCR was more sensitive than qPCR [93]. In general, the ddPCR technique with high sensitivity and specificity attracts increasing interest in its potential for clinical diagnosis and screening, and has the potential to be considered in schistosomiasis diagnosis as a complement to routine assays in schistosomiasis elimination programs.

### 4.5. Loop-Mediated Isothermal Amplification (LAMP)

The LAMP technique, which uses isothermal conditions to amplify DNA, is relatively simple, cost-effective, rapid, and more field-friendly compared with commonly used PCR-based methods [51,112]. Isothermal amplification does not require specific equipment, such as a thermocycler, electrophoresis apparatus, UV transilluminator, etc., while only a heating block or hot water bath is required for the reaction to progress [83,113,114]. The amplification results can be judged by precipitation turbidity of magnesium pyrophosphate or color reaction with the naked eye [115]. Hence, it is more suitable in resource-poor settings and grass-roots units. In addition, the four specific primers designed for six regions of target genes make the assay highly specific [113,114]. Of course, the LAMP technique has some shortcomings that need to be improved. It is complicated and time-consuming in the process of initial optimization with the use of multiple primers. Sometimes, the false-positive reaction is the fatal defect of the LAMP assay because of its high sensitivity [116,117]. Overall, the LAMP method is an extraordinary innovation trying to break through the restriction of equipment, and it has tremendous potential to apply in schistosomiasis control program for rapid screening, identification of transmission foci and environmental risk assessment.

A series of LAMP assays have been designed for schistosome-infected snail detection, schistosomiasis japonica diagnosis, and chemotherapy efficacy evaluation. The genes of CaBP (calcium-binding protein) [118,119], 28S rRNA [18,19,60], and SjR2 [120,121,122,123,124,125] were selected as the target sequence in the LAMP assay. Research has shown that the LAMP assay usually displays a higher detection rate than the conventional microscopy method for snail at different stages from 1 to 10 weeks post-infection [126]. 28S rRNA-LAMP was able to amplify the target band using DNA of a 1 day post-infection snail infected with one miracidium [18]. Nowadays, the LAMP assay has been applied to the surveillance of schistosoma infection of *O. hupensis* snail in national schistosomiasis sentinel sites in China [60,127]. In addition, a few studies have been conducted to evaluate the detection efficacy of LAMP for definitive hosts of *S. japonicum*. The SjR2-LAMP assay developed by Xu et al. was able to detect *S. japonicum* DNA in rabbit sera on the 3rd day post-infection. When LAMP was used to detect *S. japonicum* DNA in clinical serum samples (*n* = 152) from *S. japonicum*-infected patients and healthy persons, the sensitivity and specificity were 95.5% and 100%, respectively [71]. Moreover, for 47 patients after treatment 3 months, 6 months, and 9 months, the negative conversion rate of *S. japonicum* DNA in patient sera increased from 23.4% to 61.7% to 83.0%, respectively [123]. The above study demonstrated that the SjR2-LAMP method provides a useful and practical tool for the routine diagnosis and therapeutic evaluation of animals and human schistosomiasis.

### 4.6. Recombinase Polymerase Amplification (RPA)

Another isothermal amplification technology named RPA is a relatively new method that has experienced exponential growth in terms of publications, popularity, and applications since its first report in 2006 [128]. The central components of RPA mainly include DNA polymerase, DNA binding proteins, and recombinase. It is reported that RPA can operate at 37~42 °C and amplify as low as 1~10 copies of target DNA to detectable levels in less than 20 min. Therefore, the novel method is remarkable for its high sensitivity, simplicity, and extremely rapid amplification, as well as its operation at a low and constant temperature [129,130]. The RPA technique has been successfully integrated with different detection strategies, from end-point lateral flow strips to real-time fluorescent detection, among others, making this technique more user friendly, equipment-free, and facilitating the quantification of DNA [130]. In a meta-analysis of the diagnostic value of nucleic acid detection in schistosomiasis japonica, the isothermal amplification technique showed a relatively higher accuracy than the PCR-based amplification technique, and the sensitivity and specificity of the RPA method was higher than the LAMP assay [131]. However, RPA also has some disadvantages, such as the higher cost, carry-over contamination, and complicated optimization process [129,130]. Furthermore, due to the single source of RPA reagents or commercial kits, alternative products of the recombinase-aided isothermal amplification technique (RAA) have been developed in China [132,133,134,135,136,137].

The diagnostic method of RPA established for schistosomiasis japonica are concentrated after 2015, and the first retrievable literature was published in 2016 [23]. The visual detection method LFD-RPA (combination of RPA and lateral flow dipstick (LFD)) targeting SjR2 could detect 5 fg of *S. japonicum* DNA and showed no cross-reaction with other parasites. The reaction could be finished within 15~20 min at a wide temperature range (25–45 °C). Furthermore, the LFD-RPA assay performed 92.86% sensitivity (13/14), 100% of specificity (31/31) and excellent diagnostic agreement with the KK method (k = 0.947, Z = 6.36, *p* < 0.001), indicating that the LFD-RPA assay has a great potency in field application [35]. The real-time RPA (RT-RPA) targeting SjR2 gene performed 0.9 fg *S. japonicum* DNA detection limit, 100% sensitivity and specificity in detection of *S. japonicum* in stool samples from 30 infected patients and 30 healthy persons. The reaction could distinguish *S. japonicum* from other worms by measuring fluorescence using the Twista^TM^ incubator block [24]. Deng et al. tried to establish a detection method for *S. japonicum* using the SjR2 gene by RPA combined with electrochemical (EC) DNA biosensor. The RPA-EC combinational detection method also exhibited high sensitivity (0.01 fg detection limit), good specificity, and the ability to complete reaction within 30 min at 37 °C [138]. Afterwards, the RPA or LFD-RPA assay for different biomarkers of 28S rRNA and SjCHGCS19 was developed, which also proved that the technology was sensitive, specific, fast, and convenient [139,140].

## 5. Prospect of Molecular Detection Methods in Schistosomiasis Diagnosis

Unlike laboratory research, the costs for large-scale screening using the molecular detection method certainly constitute a major constraint. This is why the KK method is widely used for epidemiological surveys and recommended by WHO for surveillance and monitoring of schistosomiasis control programs [29,141]. However, in the current era featured by low endemicity of schistosomiasis, the asymptomatic cases or light infections would be missed by the KK method and MHT, resulting in significant underestimation of prevalence. There is also a consensus that diagnostic tools should be adapted when moving from morbidity control to elimination of infection [29]. It emphasizes that the accuracy of a given diagnostic technique may vary significantly with different schistosomiasis transmission levels. Antibody detection methods are indeed the most widely used nationwide in China, but this pattern of invasive sample collection may not be accepted in the future. Additionally, the detection results are prone to cause confusion and equivocal answers due to their low specificity. Therefore, in the case of reducing the cost, the molecular detection methods are greatly promising, at least in China. In fact, China is also promoting molecular detection methods in surveillance activities, such as LAMP for snail infection detection.

Among the available molecular detection methods, the methods of directly displaying results may be more suitable for the elimination stage, such as qPCR, LAMP, RPA, or RAA. Less procedures, especially casting off an additional electrophoresis step to detect PCR end-products, can be less time consuming and less labor intensive. The qPCR methods can also provide a measurement of infection intensity. The isothermal amplification methods, including LAMP and LFD-RPA, are more field-friendly for visualizing the results directly using the naked eye. The technique of RPA or RAA further reduces the reaction temperature and shortens the reaction time, which is more in line with the requirements of POCT (point of care test). However, the reagents of RPA are much more expensive than the two other methods, and the purchase of its reagents is harder than others for a source of monopoly. Overall, the above molecular detection methods should also be optimized and verified before large-scale application. For other molecular detection methods, they can be used as reference methods for laboratory testing, and are not suitable for field use. The key advantages, limitations, and relative costs of involved molecular methods are summarized in Table 3.

## 6. Chance, Challenges, and the Way Forward

The pandemic of COVID-19 is really a catastrophe for human beings; however, this control progress has promoted the improvement of detection ability for disease control units and personnel, including those engaged in schistosomiasis control, especially for primary-level staff. In a questionnaire survey conducted in 36 countries or districts among the 12 schistosomiasis-endemic provinces in China, all CDC (Center for Disease Control and Prevention) or schistosomiasis control stations participated in the local campaign against COVID-19, and the participation rate of professionals previously engaged in schistosomiasis was 84.32% (936/1110) [38]. A few schistosomiasis control agencies have become a fixed point of coronavirus detection. Moreover, 9.51% of professionals from schistosomiasis control stations participated in virus detection, and the average working time reached 53 days, remarkably promoting the practice ability of molecular detection technologies [38]. Nowadays, most schistosomiasis control stations can independently carry out molecular detection assays, including PCR, qPCR, and isothermal amplification methods. Besides, the recognition for nucleic acid detection of residents and employees is getting higher with the widespread use of nucleic acid detection methods in COVID-19 control, providing an inside track for the field application of molecular detection methods of schistosomiasis, as long as methods with high sensitivity and accuracy are developed.

Although DNA amplification-based molecular diagnostic techniques for schistosomiasis japonica truly have made gratifying progress in recent 20 years, continuous efforts are still needed to establish accurate, field-deployable diagnostics, meeting the demands of national control programs and adapting to resource-limited and low prevalence endemic settings. It should also be noted that the current variable temperature and isothermal DNA amplification techniques present several important disadvantages in real application, including complicated DNA extraction, inseparable cryogenic storage, instability, carry-over contamination, and the unfriendliness of large-scale screening [51,113,116,117]. Nucleic acid extraction is a pivotal procedure, and it may be a bottleneck in DNA detection assays since the yield and quality of DNA directly affect the outcome of the amplification procedure. Meanwhile, nucleic acid purification is considered one of the important challenges preventing molecular diagnostics adoption from reaching the field, and the extraction procedure is also often the most expensive part of DNA-based diagnosis, particularly when using commercially available extraction kits [142,143]. Hence, simplifying available DNA extraction procedures to make them more convenient and less expensive is an urgent challenge to be solved. Currently, most molecular diagnostic reagents need cold chain transportation, and even some enzymes require more stringent temperature conditions [143,144]. Although the developed logistics can partly solve the transportation problem, long-distance transportation will undoubtedly increase the cost and waste of reagents, especially in resource-limited areas and remote areas. Nowadays, there are studies focused on ready-to-use reaction mixes stored at room temperature or at 4 °C [145]. Stability is also a crucial factor in evaluating a method, especially in different conditions of carriage, storage, and experimental environments. Unfortunately, contrasting results occurred in some studies [18,71,146], suggesting that the developed approaches should be evaluated in a multicenter manner. Carry-over contamination is another nerve-wracking problem, especially for isothermal amplification techniques with higher sensitivity [51,112]. Moreover, once the contamination appeared, it was very difficult to remove it in the same lab room. Hence, it is necessary to optimize the operation procedure to adapt to the resource-limit lab. However, this optimization process is also time-consuming, complicated, and costly [116,117].

There are a set of criteria that the diagnostic methods should fulfill to be considered an ideal POCT or large-scale screening test. The criteria can be established by the acronym ASSURED, namely affordable, sensitive, specific, user-friendly, rapid, and robust, equipment free, and deliverable [147]. The isothermal amplification techniques, especially LAMP, already fulfill most of the requirements of the criteria of ASSURED. However, there is a very low probability that the isothermal amplification techniques (LAMP and RPA) can be developed to be completely equipment-free technologies; equipment dependence can be reduced and simplified to the fullest by combining with lateral flow dipsticks, microchips, and other lab-on-chip displays [148,149,150]. Actually, we have to admit that there are no significant changes in current molecular diagnostic protocols for schistosomiasis japonica, including the isothermal amplification method, although LAMP has been available since 2000.

## 7. Conclusions

Development and implementation of optimal methodologies for diagnosis is crucial in all aspects of schistosomiasis japonica control. Diagnostic tools with high sensitivity and specificity are needed as programs shift their goals from control to elimination in China [6,7]. Diagnostic technologies based on nucleic acid amplification can offset the deficiency of traditional parasitic methods. Meanwhile, the isothermal amplification techniques have made significant breakthroughs in breaking traditional laboratory boundaries by providing nucleic acid replication at constant temperatures [129]. It is gratifying that some glorious progress has been achieved, including the discovery of various biomarkers and the establishment of multiple kinds of detection techniques. Moreover, we have also tried to carry out the integration of LAMP in routine surveillance of schistosomiasis [127]. In the stage of schistosomiasis elimination, not only PCR-based detective methods but also isothermal amplification assays can be used as a vital supplement to traditional diagnostic methods of etiological and serological techniques. Certainly, simplifying and standardizing the existing molecular diagnostic methods and adjusting them to field application, especially the isothermal amplification method, are aspects that require continuous effort.

## Figures and Tables

**Table 2:** The target genes and application performance of qPCR assay for schistosomiasis japonica.

	Target Sequence	No. of GenBank Accession	Fragment Size (bp)	Detection Limit	Sensitivity (%)	Specificity (%)	Prevalence ^1^	Specimen	Year of Publication
1	NADH I	N/D ^2^	82	1 EPG	N/A ^3^	N/A	N/A	Human feces	2006 [84]
2	NADH I	N/D	75	1 EPG	N/A	N/A	5.3%	Human feces	2009 [85]
3	NADH I	N/D	82	5 EPG	100/96.83	100	11.06% of human; 24.73% of bovines	Buffalo and human feces	2018 [86]
4	NADH I	N/D	82	1 EPG	100	100	51.5%	Buffalo feces	2010 [87]
5	NADH I	N/D	82	one egg; 14 pg	95.25/94.0	100	95.45%	buffalo and Human feces; gDNA	2012 [88]
6	NADH I	N/D	82	N/A	N/A	N/A	87.50%	Bovines feces	2015 [89]
7	NADH I	N/D	82	N/A	N/A	N/A	90.2%	Human feces	2015 [90,91]
8	NADH I	N/D	82	N/A	N/A	N/A	N/A	Serum, urine and fecal of pig model	2008 [92]
9	NADH I	N/D	82	5 EPG	N/A	N/A	N/A	Buffalo feces	2009 [93]
10	NADH I	N/D	82	N/A	N/A	N/A	9.21% of rodents; 18.37% of dogs; 6.9% of goats	Rodents, dogs and goats feces	2017 [94]
11	NADH I	AF215860	75	N/A	N/A	N/A	N/A	Organs	2018 [95]
12	COX I	N/D	119	N/A	N/A	N/A	N/A	Water samples	2019 [96]
13	18S rRNA	AYl57226	81	6.15 pg	N/A	N/A	48.0%	gDNA	2008 [97]
14	18S rRNA	AY157226	81	10 fg	N/A	N/A	N/A	gDNA	2011 [98]
15	18S rRNA	AY157226.1	N/D	20 fg	N/A	N/A	N/A	gDNA	2011 [99]
16	18S rRNA	FJ176682	156	4.3 × 10^2^ copies plasmid; 0.4 fg gDNA; 10 EPG; one cercaria in 10 pooled snails;	N/A	N/A	N/A	Plasmids; gDNA; mice feces; snail	2013 [100]
17	18S rRNA	AY157226	280	10 fg	N/A	N/A	N/A	gDNA	2015 [101]
18	ITS 2	U22167	192	1.42 × 10^2^ copies plasmid; 10 pg gDNA; 10 EPG;	100	100	N/A	Snail and mice feces	2011 [102]
19	SjR2	AF412221.1	N/D	2 pg	N/A	N/A	N/A	gDNA	2011 [99]
20	SjR2	AF412221	N/D	44.7 copies plasmid	N/A	N/A	N/A	Plasmids and sera of rabbit	2014 [103]
21	SjR2	N/D	N/D	N/A	N/A	N/A	N/A	Water samples	2021 [104]
22	SjCHGCS20	FN356222.1	N/D	N/D	98.74	100	8.33	Plasma of goat	2020 [22]
23	SjCHGC08270	AY812553	85	half of one cercaria	93.75	N/D	N/A	N/A	2008 [20]
24	SjCHGC08270	AY812553	85	one cercaria	N/A	N/A	6.48	Water samples	2011 [105]
25	Sjrh1.0	U92488.1	N/D	2 fg	N/A	N/A	N/A	gDNA and sera of mice	2011 [99]
26	Sjrh1.0	U92488.1	N/D	2 fg	N/A	N/A	N/A	gDNA and water samples	2011 [106]

^1^ Prevalence: no. positive/no. examined; ^2^ N/D: Non-disclosed; ^3^ N/A: Non-applicable.

**Table 3:** Advantages, limitations, and prospects of large-scale application of different DNA diagnostics in China [51]. (The copyright permission of the Table 3 in the cited reference with modified form has been obtained from corresponding author of Professor Don McManus).

Method Type	Advantages	Limitation	Instrument Cost *	Reagents Cost *	Prospect of Large-Scale Application **
cPCR	Low cost and simple among the molecular detection methods; Can be multiplexed based on different size domains of the gene.	Requires post-PCR processing causing it to be more time consuming and labor intensive;	$	$	∆
nPCR	Improved the sensitivity and specificity for using two sets of primers	Relatively complicated initial optimization process; More time consuming and labor intensive than two rounds of cPCR amplifications; Prone to contamination with amplified PCR products	$	$	∆
qPCR	Higher sensitivity and specificity when probes are used; No post PCR processing and less time consuming and less labor intensive compared to cPCR and nPCR; Can quantify the amount of amplicons; Lower potential laboratory contamination	Relatively complicated initial optimization process; Requires triplicate reactions to improve the accuracy of final calculations	$$	$	∆∆
ddPCR	Higher sensitivity, specificity, specifically when probes are used; Can quantify the amount of amplicons (absolute quantification); Lower potential laboratory contamination	Requires specific and expensive machinery for the initial establishment; Relatively time consuming and complicated initial optimization process	$$$	$$	∆
LAMP	Less equipment required; Can visualize the end products directly using naked eye	Relatively time consuming and complicated initial optimization process; Prone to carryover contamination	-	$	∆∆∆
RPA	Less equipment required; End products can be visualized on a chip/lateral flow device; Has great potential to be developed as a point of care diagnostic tool	Relatively complicated initial optimization process; Prone to contamination	-	$$$	∆∆

* The cost of the instrument and reagents is given as a relative scale to each other. Although the same conformity is used, the price of the instrument and reagents is not the same. $:—low, $$—moderate, $$$—high. ** It represents the possibility that it can be used as a tool for on-site screening and surveillance in the future: ∆—low, ∆∆—moderate, ∆∆∆—high.

## Data Availability

Not applicable.
